# Imaging black holes: a VLBI success story

**DOI:** 10.1007/s10509-026-04598-w

**Published:** 2026-06-30

**Authors:** J. Anton Zensus

**Affiliations:** Max-Planck-Institut für Radioastronomie, Postfach 2024, 53121 Bonn, Germany

**Keywords:** Supermassive black holes, Very long baseline interferometry, Event Horizon Telescope, Relativistic plasma jets

## Abstract

Advances in Very Long Baseline Interferometry (VLBI) have enabled, for the first time, horizon-scale images of the two most accessible supermassive black hole candidates: M87* at the centre of the elliptical galaxy Messier 87 and Sgr A* at the centre of the Milky Way. These images reveal ring-like emission surrounding central brightness depressions whose angular sizes are consistent with the black-hole signatures predicted by general relativity. The theoretical framework and early predictions of observable black hole signatures, developed from the 1970s onward, established the scientific motivation and defined the instrumental requirements for this endeavour. Achieving the required angular resolution and sensitivity demanded transformational enhancements to existing VLBI arrays, pursued along two complementary paths: space VLBI, extending baselines beyond the Earth, and mm-VLBI, observing at the shortest accessible radio wavelengths from the ground. The latter strategy, realised through the addition of the Atacama Large Millimeter Array to a global network of mm-wavelength telescopes, advances in receiver technology, data processing, and image reconstruction methods, and the formation of an international collaboration, yielded the first images of the predicted black hole shadows. This established the observational foundation for a broad programme of follow-up studies.

## Introduction

The first horizon-scale images of the supermassive black hole (SMBH) candidates M87* and Sgr A*, obtained by the Event Horizon Telescope (EHT) Collaboration (2019a, 2022), represent a landmark achievement in observational astronomy. They are the product of a sustained global effort to advance Very Long Baseline Interferometry (VLBI) in angular resolution, sensitivity, and imaging fidelity over more than four decades. These developments were directed towards probing the central regions of Active Galactic Nuclei (AGN) at angular scales previously inaccessible to any observational technique. The theoretical predictions underpinning these images predate their realisation by nearly half a century, reflecting the extraordinary instrumental demands of the endeavour.

The central structure of the galaxy Messier 87, named M87*, is revealed in the EHT image as a bright emission ring surrounding a central shadow^1^ consistent with theoretical predictions, providing compelling observational evidence for the presence of a supermassive black hole.^2^

Two years elapsed between the 2017 observing campaign and the publication of the M87* image. A further three years passed before the publication of the image of Sagittarius A* (Sgr A*), the black hole at the centre of the Milky Way (EHT Collaboration 2022), a delay attributable in part to the greater complexity of the Sgr A* data and in part to disruptions caused by the Covid-19 pandemic.

These results represented the culmination of decades of coordinated technical and theoretical development. This paper^3^ describes the efforts of the international VLBI community towards the angular resolution, sensitivity, and image quality required to image SMBHs, and more broadly towards advancing understanding of compact radio sources and the relativistic jets emanating from AGN. The development of mm-VLBI is reviewed in greater technical detail in Boccardi et al. (2017) and a comprehensive treatment of relativistic jets from active galactic nuclei is provided by Blandford et al. (2019). The paper does not address the major applications of VLBI to astrometry, geodesy (Schuh and Behrend 2012), or the physics of the circumstellar and interstellar medium (e.g., Reid et al. 1980).

## General relativity and massive astrophysical objects

In 1919, just after the end of World War I, British astronomers Frank Dyson and Arthur Eddington organised an expedition to measure the deflection of the apparent positions of stars near the Sun’s limb during the solar eclipse on 29 May 1919, as predicted by Albert Einstein’s Theory of General Relativity (GR; Einstein 1916). The expedition took place almost exactly 100 years before the publication of the EHT papers reporting the first image of M87*.

Einstein’s approximation for the light deflection is

1 $$ \Delta \varphi \sim 1.75^{\prime \prime } M / b, $$

where $M$ is the mass of the deflecting body in solar masses M_⊙_, and $b$ is the radial distance of the deflected light ray in solar radii. This approximation can also be applied to compact objects such as the subsequently postulated neutron stars and black holes.

The Dyson-Eddington expedition (Dyson et al. 1923)^4^ provided the first widely recognised observational support for Einstein’s theory, offering a descriptive framework for astrophysical phenomena beyond the reach of classical theory. The full implications of the theory for astrophysical phenomena were not yet appreciated; it was not until the late 1950s and 1960s that astrophysicists became fully aware of its power to describe newly discovered phenomena such as quasars and pulsars.

The discovery of pulsars (Hewish et al. 1968) and their identification as compact rotating neutron stars (Gold 1968) opened a new regime for testing relativistic gravity beyond the solar system. This became especially clear with discovery of the first binary pulsar by Russell Hulse and Joseph Taylor, which provided the first indirect evidence for gravitational waves: the orbital period was decreasing at exactly the rate predicted by GR for energy carried away as gravitational radiation (Hulse and Taylor 1975). The discovery earned them the 1993 Nobel Prize.

In 1939, Robert Oppenheimer and Hartland Snyder provided the first relativistic treatment of gravitational collapse, describing the dynamical process by which a sufficiently massive star contracts without limit, in what is now understood as the formation of a black hole (Oppenheimer and Snyder 1939). The mass range of stellar black holes is typically expected to lie between a few and a few tens of solar masses. Following a suggestion by Webster and Murdin (1972) that Cyg X-1 contained a massive and invisible companion, Reid et al. (2011) determined the trigonometric distance to Cyg X-1, enabling a mass estimate above the neutron-star range and thus implying a stellar black hole. A gravitational wave signal captured by the Laser Interferometer Gravitational-Wave Observatory (LIGO) provided evidence for the merger of two stellar black holes (Abbott et al. 2016). A binary system comprising a pulsar orbiting a 2.1–2.7 M_⊙_ object, which may well be a stellar black hole, was recently reported (Barr et al. 2024). Primordial black holes with sub-stellar masses, remnants of the Big Bang, have been theorised (Zel’dovich and Novikov 1967), though their detection would present extraordinary observational challenges.

**Fig. 1:**
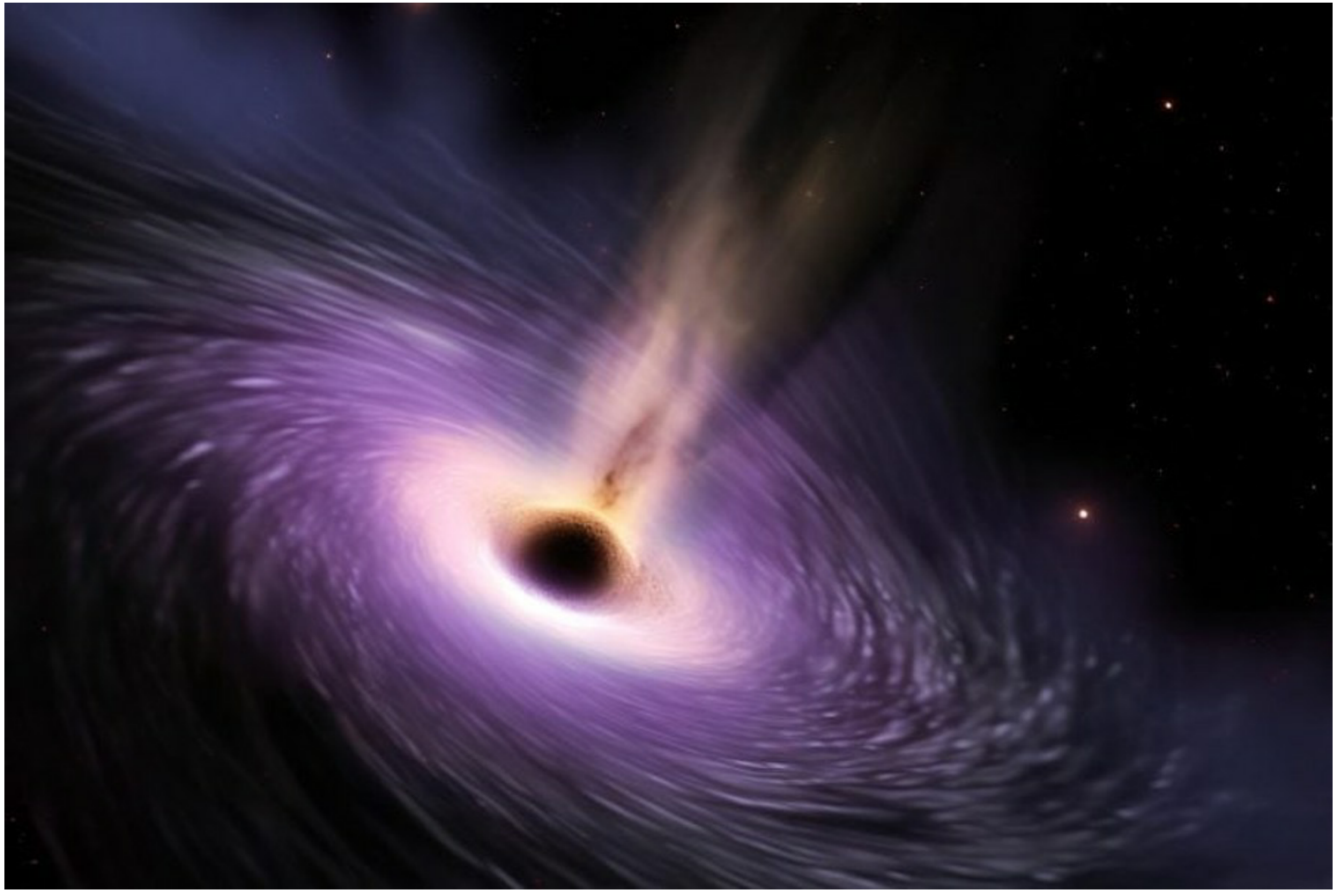
Artist’s impression of an active galactic nucleus, showing a central SMBH surrounded by an accretion disk and launching a pair of relativistic plasma jets. Credit: Rusen Lu (Chinese Academy of Science)

Following Maarten Schmidt’s identification of the quasar^5^ 3C273 as a redshifted extragalactic radio source (Schmidt 1963), SMBHs with masses exceeding $10^{5}$ M_⊙_ were suggested to explain the nature of such quasars (Fig. 1). The physical process of energy release was described in terms of the conversion of surrounding mass into energy in 1964 by Edwin Salpeter (Salpeter 1964), while in 1969 Donald Lynden-Bell proposed that such a mass accretion should result in an accretion disk surrounding the central massive object (Lynden-Bell 1969). The prospect of VLBI observations as a means of directly testing the accretion disk hypothesis by determining the size of a central black hole was explicitly identified as a critical observational goal (Lynden-Bell and Rees 1971).

Theoretical studies (e.g., Blandford and Rees 1974) explained the formation of relativistic plasma jets ejected by the accreting system around a massive object (see Blandford et al. 2019).

**Fig. 2:**
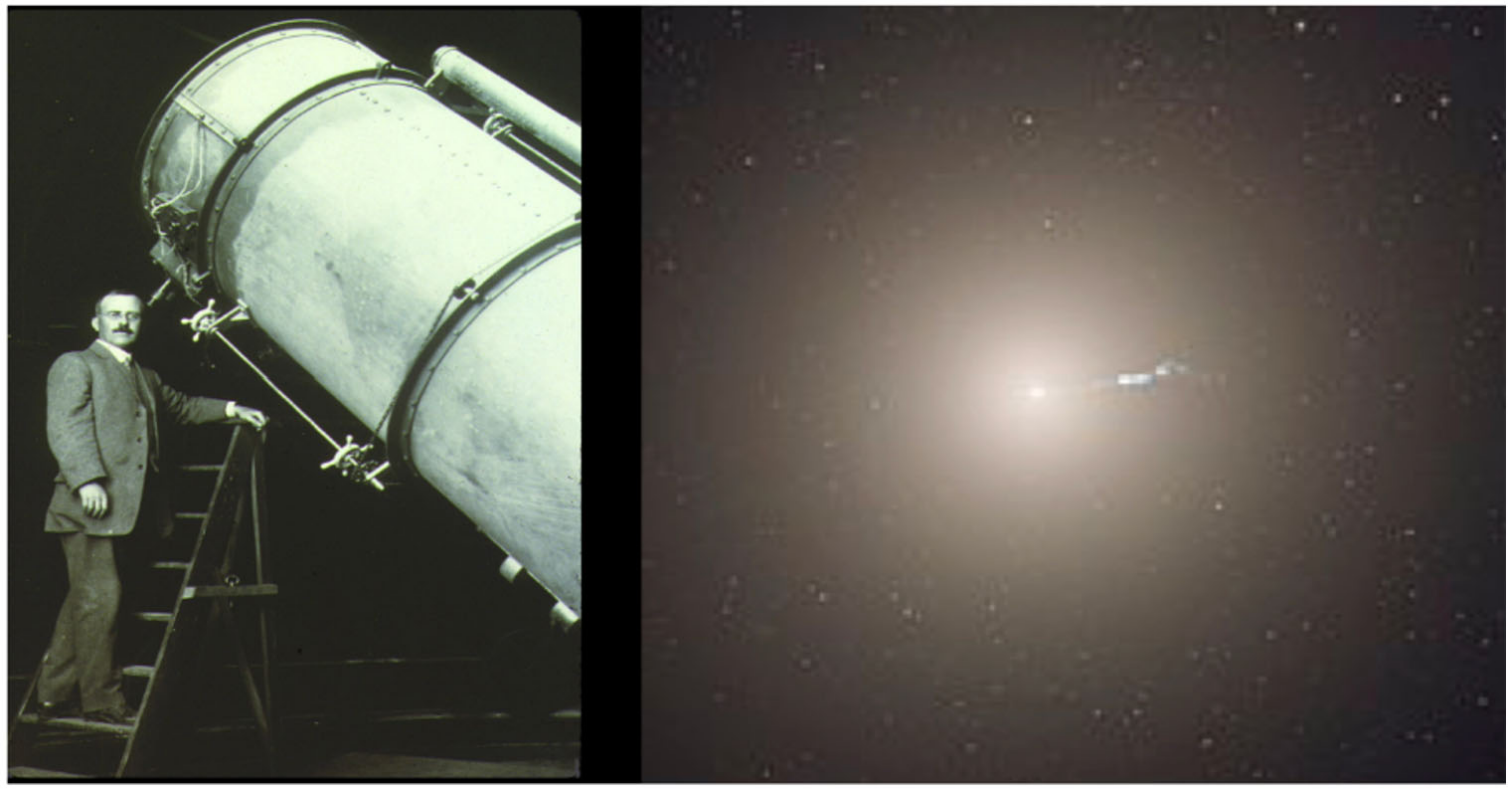
Photographic image of the elliptical nebula Messier 87 published by Heber Curtis in 1918, showing the ‘curious straight ray’ that he described as ‘apparently connected with the nucleus by a thin line of matter’ (Curtis 1918). This is the first recorded observation of a relativistic jet

Such a plasma jet had already been observed earlier. About the same time as the Dyson-Eddington expedition, in 1918, the US astronomer Heber Curtis published a photographic image of the “elliptical nebula” Messier 87 (see Fig. 2) and noticed a “curious straight ray... apparently connected with the nucleus by a thin line of matter” (Curtis 1918). The nature of objects such as M87 remained a subject of debate (Shapley and Curtis 1921) until redshift measurements and distance determinations revealed their extragalactic origin (Hubble 1929). Curtis’s observation of the jet in M87, unlike the near-contemporaneous Dyson-Eddington result, attracted little attention for several decades. With an adopted distance of $16.8 \pm 0.8$ Mpc (see EHT Collaboration 2019a) it became clear that the “line of matter” observed by Curtis must have enormous linear dimensions, raising the question of the origin of such a coherent structure, which is now understood to be a relativistic jet.

Dynamical considerations provided further supporting evidence for the existence of very massive dark objects in the centres of galaxies. For example, as early as 1978, a central mass of $5 \times 10^{9}~\mbox{M}_{\odot}$ was derived from analysing the radial velocity distribution of spectral line features as a function of distance to the very centre of M87 (Sargent et al. 1978). Rapid variability at optical and higher photon energies provides additional evidence for large amounts of energy being produced on small linear scales (Rees 1966; see also Wagner and Witzel 1995).

There was also early evidence for a central black hole in our own Galactic Centre, although the central region is found to be less active than the more luminous SMBHs (see Witzel et al. 2018). In visible light, the Galactic Centre is unobservable because of extinction of the order of 30 magnitudes (Scoville et al. 2003). High-resolution radio astronomical observations revealed a point-like source named Sgr A* (Balick and Brown 1974), which appears stationary at the centre of the Milky Way (Reid and Brunthaler 2004, 2020). Eckart and Genzel (1996) concluded from dynamical considerations the existence of a mass of about $2.5 \times 10^{6}~\mbox{M}_{\odot}$ within a radius of 0.015 pc or $0.4''$, providing strong evidence for a black hole in the centre of our Galaxy, even though the Schwarzschild radius of such a putative black hole would be many orders of magnitude smaller than the derived limit. Reinhard Genzel and Andrea Ghez, working independently with their respective groups, refined this mass estimate through long-term observations of closed stellar orbits around Sgr A*, with pericentre distances down to 100 AU, yielding a central black hole mass of $4.3 \times 10^{6}~\mbox{M}_{\odot}$ (Ghez et al. 2008; Gillessen et al. 2009; GRAVITY Collaboration 2019). These measurements have since been refined further using near-infrared interferometry with GRAVITY, which detected relativistic deviations from Keplerian motion at the periapsis passage of star S2. This provides independent confirmation of strong-field general relativity in the immediate vicinity of Sgr A* (GRAVITY Collaboration 2019). This body of evidence was recognised with the award of the 2020 Nobel Prize to Genzel and Ghez for the discovery of a supermassive compact object at the centre of the Galaxy.

## Predictions of measurable structure around black holes

**Fig. 3:**
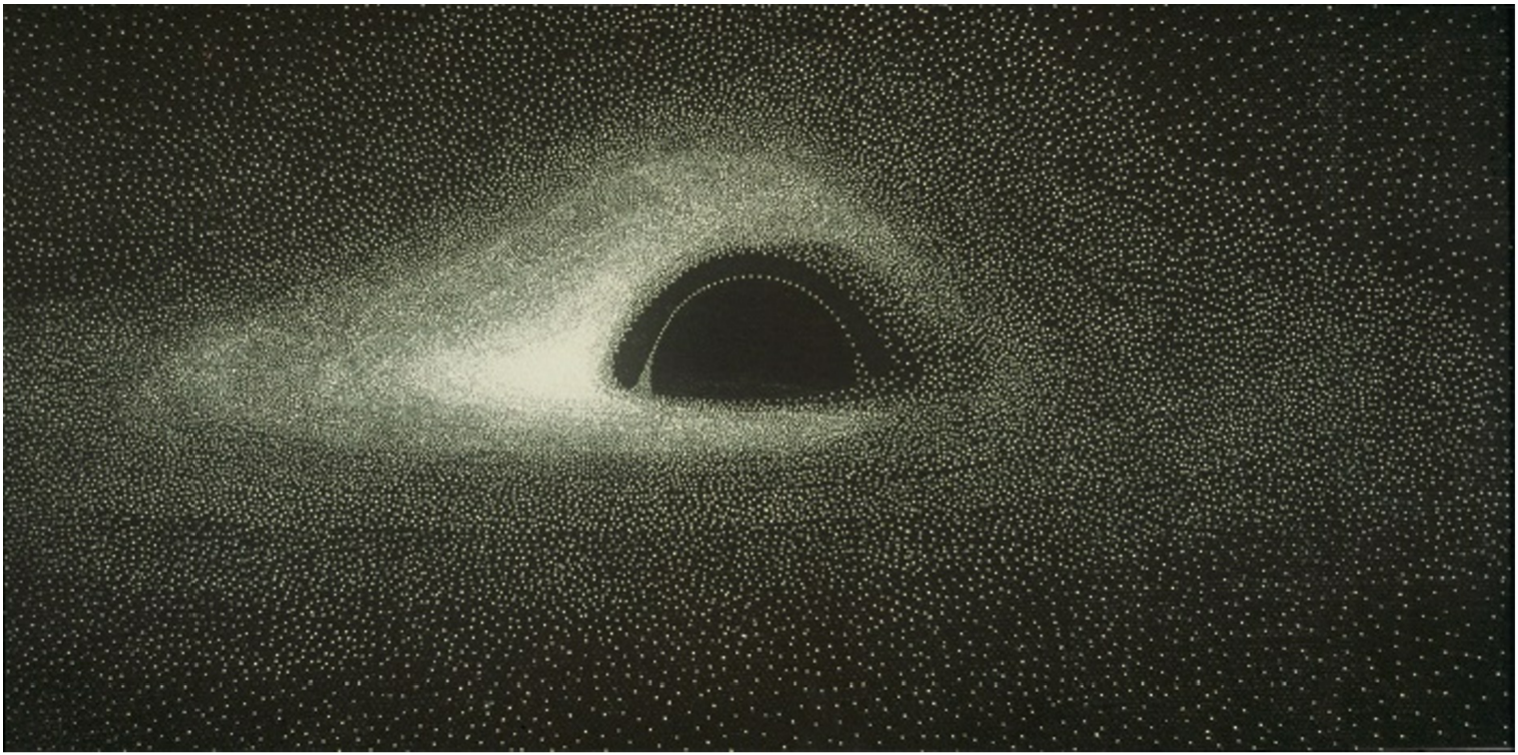
The first detailed numerical simulation of the appearance of a black hole with a thin accretion disk, computed by Jean-Pierre Luminet (1979). The dark central region is the black hole shadow, i.e. the set of directions on the observer’s sky from which photons are captured by the black hole rather than reaching the observer. Its boundary is determined by unstable photon orbits, and its apparent size (diameter) is approximately 5.2 times the Schwarzschild radius. It is surrounded by strongly lensed emission, including contributions from higher-order images associated with unstable photon orbits. The asymmetry of the surrounding emission reflects the Doppler boosting of the relativistically rotating disk. Lacking graphical display capabilities, Luminet produced the image manually from numerical output (Luminet 2019)

By the 1970s, computational capabilities had advanced sufficiently to permit ray-tracing techniques for studying gravitational lensing effects on electromagnetic radiation from black hole accretion disks. For an overview of the early developments towards black hole imaging, from 1972 to 2002, see the account by Jean-Pierre Luminet (2019). As early as 1972, Chris Cunningham and his Ph.D. advisor James Bardeen described the apparent positions of a star orbiting a Kerr black hole (Cunningham and Bardeen 1972), and Cunningham was the first to describe, in his thesis, the effect of a Kerr black hole with a thin accretion disk on an observer (Cunningham 1975). Working independently, Luminet published the first detailed calculated image of the distortion of an accretion disk by a black hole in 1979 (Fig. 3). This simulation showed the image of the accretion disk strongly distorted by gravitational lensing, with the dark shadow of the black hole at its centre. In this bolometric simulation, part of the shadow is covered by the part of the opaque accretion disk that lies between the black hole and the observer.^6^ According to Luminet (see also von Laue 1921), the shadow of the black hole has a radius of

2 $$ r_{\mathrm{shadow}} = 3 \sqrt{3} r_{g}, $$

where $r_{g}$ is the gravitational radius, i.e. half the Schwarzschild radius (Schwarzschild 1916), which in units convenient for SMBHs is

3 $$ r_{g} / \mathrm{AU} = 0.00987\ M /( 10^{6}\ \mathrm{M}_{\odot} ). $$

In the 1980s and 1990s there was growing interest in simulating the appearance of enshrouded black holes of various spins and geometries (Fukue and Yokoyama 1988; Jaroszynski et al. 1992; Hollywood and Melia 1997), including animations (Luminet 2014).

While previous simulations were primarily concerned with the theoretical appearance of the shadows of rotating or non-rotating black holes, Heino Falcke and collaborators (Falcke et al. 2000) addressed the specific observational case of Sgr A* at short mm and sub-mm wavelengths, and found that the derived diameter of the shadow begins to resolve at the longest terrestrial VLBI baselines. In fact, from Eqs. (2) and (3) the diameter of the shadow in microarcseconds^7^ becomes

4 $$ \theta _{\mathrm{shadow}} ( \mu \mathrm{as} )= 0.103 \frac{M /( 10^{6}~\mathrm{M}_{\odot} )}{D /( \mathrm{Mpc} )}, $$

where $D$ is the distance to the source. Applying these relations to the best available mass and distance estimates for the most promising SMBH candidates yielded comparable predicted shadow diameters for Sgr A* and M87* (approximately 50 and 40 microarcseconds, respectively). These predictions demonstrated the feasibility of direct black-hole imaging with mm-VLBI and helped establish Sgr A* and M87* as the principal targets for the instrumental and organisational developments described in the following section.

## Very Long Baseline Interferometry and the quest for microarcsecond-scale imaging

Radio astronomy has provided a fundamentally new observational window on the universe since the discovery of cosmic radio waves from the Galaxy in the 1930s (Jansky 1933; see Kellermann and Bouton 2023). Single-dish radio telescopes, however, are severely limited in angular resolution: even the largest facilities, such as the Effelsberg 100-m telescope in Germany and the Green Bank Telescope in West Virginia, achieve resolution comparable only to that of the human eye. The development of interferometric techniques, combining spatially separated radio telescopes, overcame this limitation and enabled angular resolution sufficient for size and structure determinations of compact sources. Martin Ryle’s foundational work on aperture synthesis (Nobel Prize in Physics, 1974) established the theoretical and practical basis for high-fidelity radio imaging (Ryle and Hewish 1960), and by the early 1960s it had been recognised that replacing the physical connections between array elements with independent atomic clocks, frequency standards, and data recorders would permit baseline lengths, and therefore angular resolutions, far beyond those achievable with connected interferometers (Kellermann and Cohen 1988; Moran 1998).

These early developments in VLBI were largely driven by the scientific challenge posed by enigmatic compact radio sources associated with quasars, whose extreme variability and apparent compactness demanded new observational tools. The discovery of apparent superluminal motion in several such objects was a particularly compelling motivation, as this phenomenon can be explained by relativistic motion at a small angle to the line of sight within collimated jets extending from parsec to kiloparsec scales (Whitney et al. 1971; Cohen et al. 1971; see also Zensus and Pearson 1987, 1990).

**Fig. 4:**
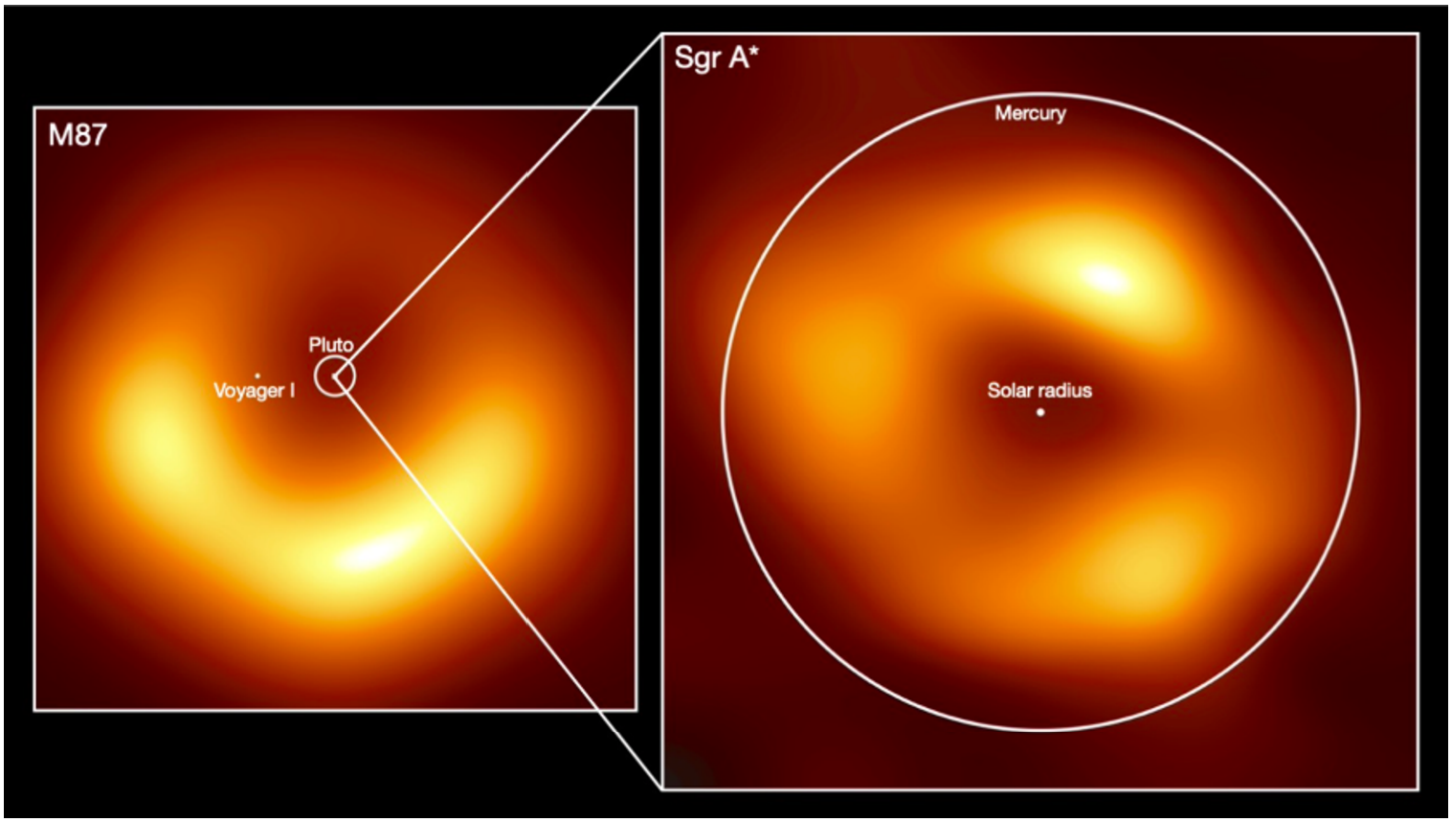
EHT images of the millimetre emission surrounding the black-hole shadow of M87* and Sgr A*, shown on the same angular scale. The indicated Pluto and Mercury orbits highlight the extreme difference in linear scale (image montage based on Medeiros 2022)

By the late 1990s, the theoretical predictions for black hole signatures had matured substantially and the consensus in the field held that supermassive black holes surrounded by luminous accretion material were ubiquitous in extragalactic nuclei and the Galactic Centre (e.g., Kellermann et al. 1977).^8^ However, achieving direct imaging required overcoming two fundamental obstacles: the angular resolution needed exceeded the capabilities of existing VLBI arrays, and the sensitivity had to improve by orders of magnitude to detect such faint structures. These twin challenges made clear that incremental technical advances would be insufficient. It had been recognised that the integration of a large millimetre array would be necessary to ‘push the detection limit and imaging capabilities of a global VLBI interferometer by 1-2 orders of magnitude’ (Krichbaum 1996). This requirement would eventually be met by the Atacama Large Millimeter Array. The scale of this undertaking exceeded the resources and infrastructure of any single institution, making an international consortium an organisational necessity. The EHT Collaboration provided the institutional framework through which the first black hole images were achieved (Fig. 4). The two images illustrate a physically significant coincidence: although M87* lies three orders of magnitude farther away than Sgr A* (which is at a distance of 8.2 kpc), its mass is correspondingly three orders of magnitude greater, yielding comparable angular sizes on the sky. The bright annular structures visible in both images are produced by synchrotron-emitting plasma in the strongly lensed region around each black hole. These structures are related to, but should not be directly identified with, the mathematically narrow photon ring formed by higher-order photon trajectories (Johnson et al. 2020). The central brightness depression corresponds to the black hole shadow, whose characteristic angular diameter is primarily determined by the mass-to-distance ratio and is largely insensitive to the details of the accretion flow. The observed angular sizes and morphologies are therefore consistent with the expectations of general relativity for black holes with the independently determined masses and distances of M87* and Sgr A*. This quantitative agreement indicates that the mass determinations are robust with respect to uncertainties in the accretion geometry and viewing angle.

The two images also demonstrate the versatility of the VLBI technique in characterising SMBHs across a wide range of physical parameters and activity levels. In the case of M87*, image analysis yields a black hole mass of $6.5 \pm 0.7 \times 10^{9}~\mbox{M}_{\odot}$ (EHT Collaboration 2019c), consistent with prior stellar-dynamical measurements, including the earliest dynamical estimate of $5 \times 10^{9}~\mbox{M}_{\odot}$ from stellar velocity dispersion (Sargent et al. 1978). For Sgr A*, the angular size of the observed shadow is in precise agreement with the mass and distance established by decades of infrared stellar-orbit measurements. This result substantially strengthens the black hole interpretation.

### Angular resolution

**Fig. 5:**
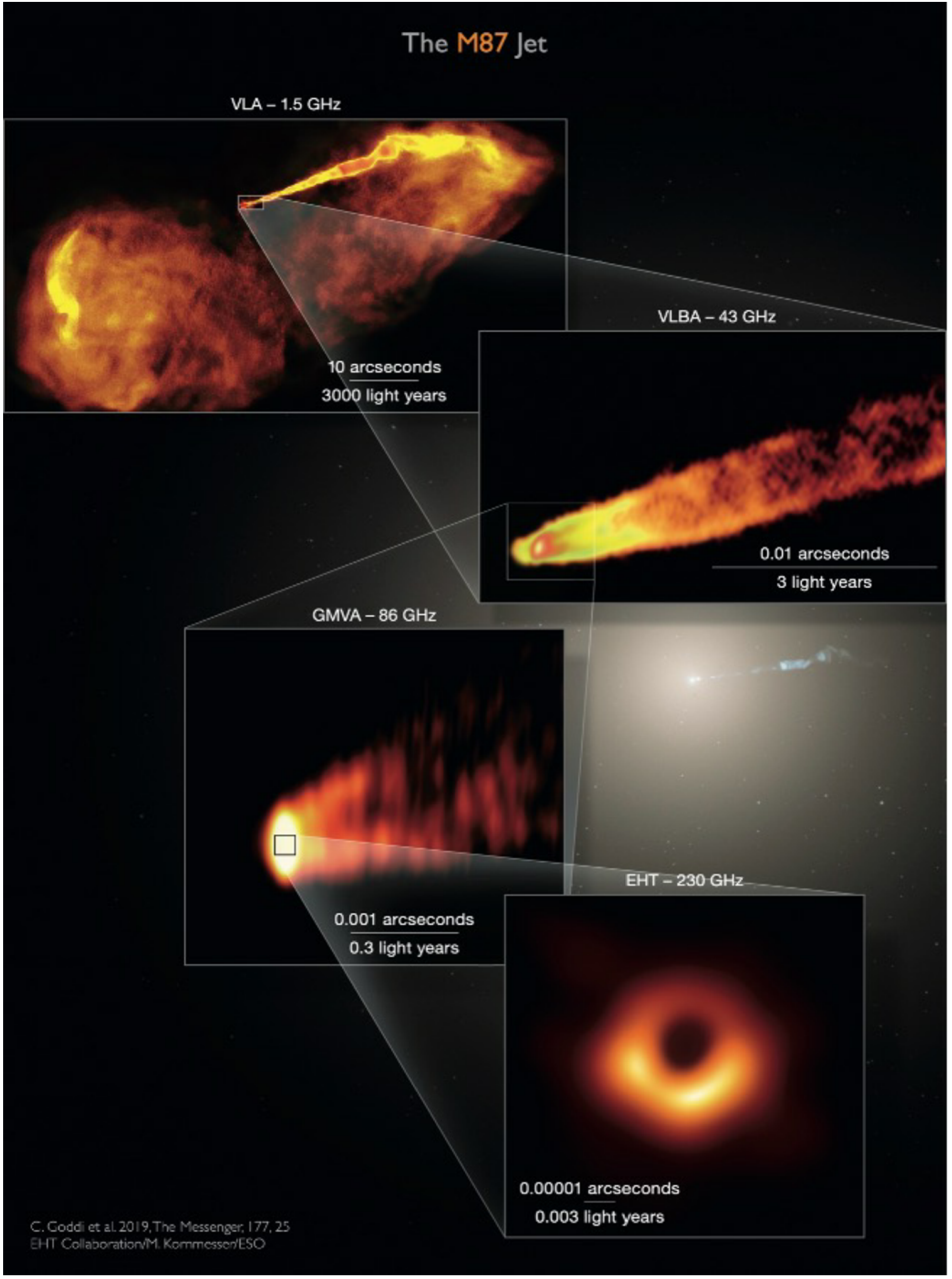
Interferometric radio observations can obtain a much higher angular resolution than even space-based optical imaging, as the example of M87* shows. The wide field of the Very Large Array (VLA) shows extended radio emission arising from the plasma jet of M87*. While the jet’s central spine is sharply resolved by the Hubble Space Telescope, HST, (grey-scale image in the background), only VLBI observations, in particular in the mm range, can resolve details a factor of more than 10^3^ smaller than in the optical. Even at a relatively long wavelength of 18 cm, VLBI has a ten times better resolution than the HST (see, e.g., the image in Reid et al. 1989). Credit: VLA, VLBA, GMVA, and EHT images compiled by Goddi et al. (2019) and references therein; HST image: NASA/ESA; montage: C. C. Goddi, Z. Younsi, J. Davelaar/M. Kornmesser/ESO

Resolving the signatures of even the most promising SMBH candidates in imaging requires an angular resolution of 20 microarcseconds or better.^9^ This is substantially beyond the capabilities of optical telescopes and single-dish radio telescopes, whose resolution is limited to about one arcminute. Radio interferometers, antenna arrays with multiple telescopes connected by cables or radio links, overcome this limitation. The VLBI method uses independent radio telescopes typically separated by thousands of kilometres, often on different continents, synchronised by atomic clocks. This effectively creates a virtual telescope up to the size of the Earth. Employing the aperture synthesis method, such observations provide the highest resolution in astronomy available today. Each interferometer baseline (an antenna-antenna pair at a given projection towards the source) measures at a given time a value of the complex interferometer visibility function, which is related to the source’s brightness distribution on the sky by a Fourier transform. The Earth’s rotation allows some of the missing Fourier components to be filled in, resulting in a relatively clean point-spread function (i.e., the image one would derive for a point source, usually referred to as the “beam” in the interferometer image). The state of the art in the mid-1990s was defined by VLBI at cm-wavelengths using intercontinental baselines. A good example is the comparison of optical and radio observations of the jet structure in M87 (Fig. 5). During that time such VLBI observations at cm wavelengths were routinely performed in several international networks, such as the European VLBI Network EVN (see Porcas 2011) and the US Very Long Baseline Array (VLBA; Napier et al. 1994), with the VLBA alone and with joint global operations achieving imaging resolutions below one milliarcsecond at the shortest cm wavelengths. This established full-Earth baseline interferometry as a mature technique and defined the resolution benchmark that mm-VLBI would need to surpass by a further order of magnitude.

Observations at shorter millimetre wavelengths presented a distinct technical frontier. These were not yet routine and were limited to a small number of antennas capable of operating in this regime. Additional telescopes required identification and technical preparation before integration into mm-VLBI arrays (e.g., the IRAM telescopes in Europe). As a result, mm-wavelength observations approached sub-milliarcsecond resolution, but often with elongated beams due to the limited range of North-South baselines, and with significant sidelobe levels due to the small number of array elements and their uneven distribution on Earth. Experiments at 3.5 mm achieved nominal resolutions of a few hundred microarcseconds, leaving approximately an order-of-magnitude improvement in angular resolution to be achieved before direct black-hole imaging became feasible (e.g., Krichbaum et al. 1997). At that time, mm-VLBI at a wavelength of 1.3 mm was still limited to the brightest sources (e.g., Padin et al. 1990) and mere “fringe-detections” of fainter AGN (Greve et al. 1995; Krichbaum et al. 1997).

As with the early development of cm-VLBI, progress at 1 mm wavelength initially depended on groundbreaking contributions from relatively small groups of specialists at observatories with suitable antennas and receiving equipment, advancing the field through incremental collaborative technical development. Early millimetre-VLBI experiments were largely confined to continental baselines. However, it became clear that transcontinental baselines at the shortest millimetre wavelengths were essential and that consolidating regional efforts into a unified global array was necessary to achieve the required sensitivity and coverage (Doeleman et al. 2002; Krichbaum et al. 2002). This recognition, together with promising precursor results, including the initial detection of event-horizon-scale structures in Sgr A* (Doeleman et al. 2008; Lu et al. 2018) and the resolution of jet-launching structure in M87 (Doeleman et al. 2012; see also EHT Collaboration 2019a), provided the impetus for the formation of the EHT Collaboration, with the explicit scientific goal of obtaining the first images of supermassive black holes.

A complementary initiative was the establishment of the Global Millimeter VLBI Array (GMVA; see Boccardi et al. 2017; Ros et al. 2024), which operates at 3 mm and provides the broader astronomical community with open-access imaging-quality mm-VLBI experiments, probing the black hole environment at an intermediate angular scale between cm-VLBI and the 1 mm EHT.

Since angular resolution is determined by both the observing wavelength and the maximum baseline length (Eq. (5)), two complementary strategies were pursued to achieve the resolution required for black hole imaging: reducing the observing wavelength to the millimetre regime, and extending baselines beyond the diameter of the Earth through space VLBI. Both avenues were explored by collaborating groups working in parallel.

The resolution of an array is defined by the observing wavelength and by the largest separation, b, of its antennas. In convenient units:

5 $$ \theta ( \mu \mathrm{as} )\sim 206\lambda ( \mathrm{mm} )/ b ( 1000 \,\mathrm{km} ). $$

Following earlier test observations, the first successful space VLBI images were achieved by the Japanese HALCA orbiting radio telescope observing together with radio telescopes on the ground, realising baseline lengths of about 30,000 km at a shortest observing wavelength of 1.3 cm (Hirabayashi et al. 1998) and improving the angular imaging resolution from VLBI to unprecedented ∼0.3 milliarcseconds, enabling improved studies of the central jet structures, particularly for stronger quasars and blazars (e.g., for 3C273, Lobanov and Zensus 2001; Fig. 7 left). In addition, the measurements of extreme brightness temperatures exceeding theoretical limits strengthened the evidence for the presence of highly beamed relativistic jets.

**Fig. 6:**
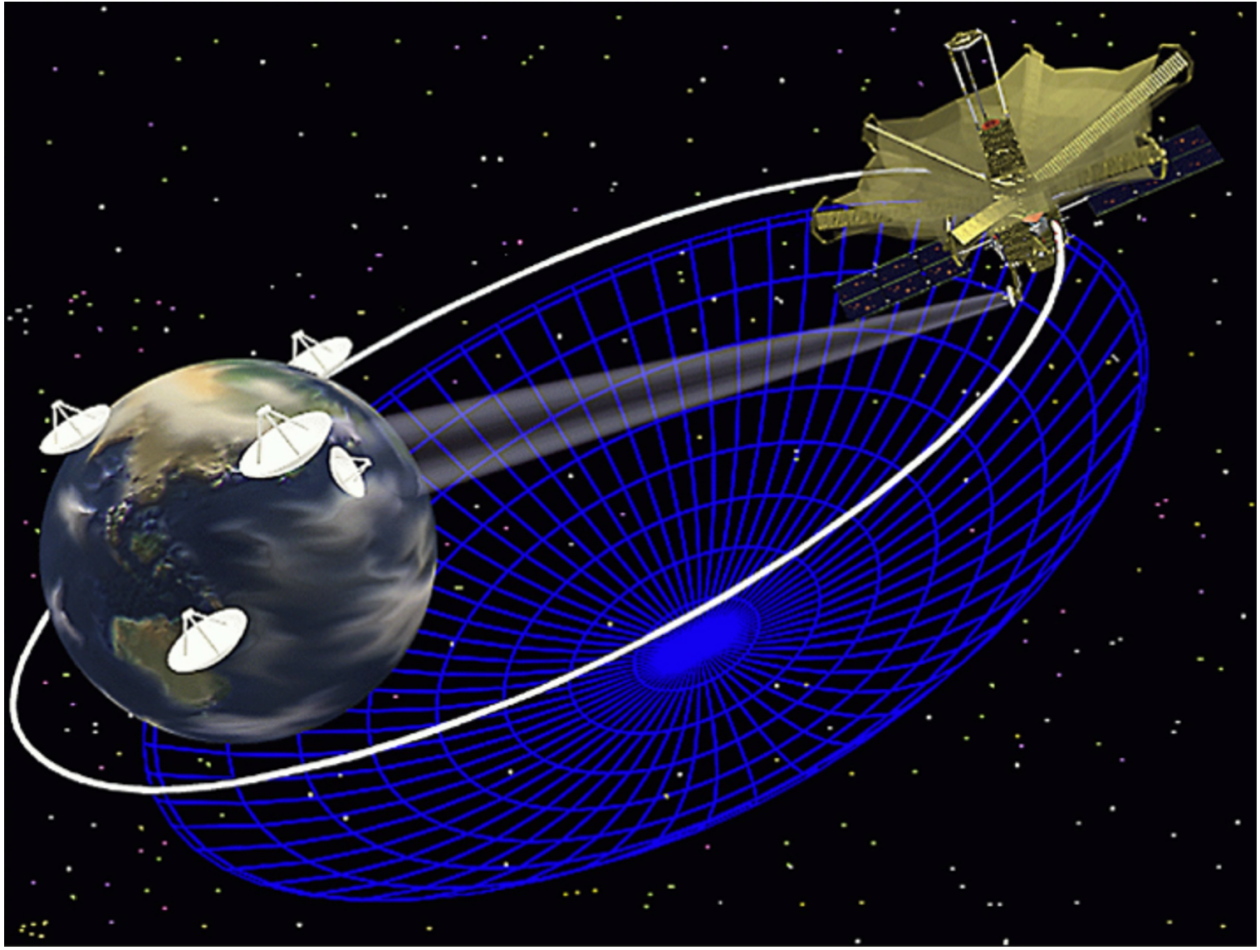
Artist’s impression of the RadioAstron space observatory, a 10-m antenna operating between 2011 and 2019 in a highly eccentric Earth orbit with an apogee of approximately 350,000 km. Co-observing with large ground-based telescopes, RadioAstron achieved imaging resolutions down to 7 microarcseconds, the highest angular resolution attained in astronomy

In 2011, the Russian RadioAstron mission (Fig. 6) successfully launched a space-borne 10-m antenna in an orbit with an apogee of nearly the Moon’s distance and also operating at wavelengths down to 1.3 cm (Kardashev et al. 2012). Despite the relatively small size of the space antenna, sensitive imaging observations became possible by co-observing with large-collecting-area telescopes on Earth (e.g., the Effelsberg 100-m telescope and the Green Bank Telescope), since the signal-to-noise ratio for a given baseline depends on the geometric mean of the collecting areas of the participating array elements. Accordingly, by integrating RadioAstron into an array of terrestrial radio telescopes, unprecedented imaging resolutions down to 7 microarcseconds could be achieved (e.g., Gómez et al. 2022), the highest ever obtained in astronomy.

**Fig. 7:**
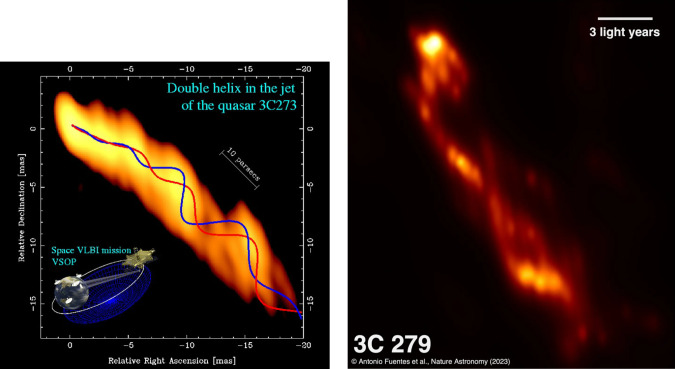
Left: The VSOP space VLBI image of the relativistic jet in the quasar 3C 273 was observed at a wavelength of 6 cm. The HALCA 8-m space antenna was combined with the VLBA and the Effelsberg 100-m telescope. The image reveals a double helical structure within the jet, representing the first direct observational evidence for Kelvin-Helmholtz plasma instability in an extragalactic relativistic jet, on scales of up to 300 parsecs from the nucleus. Credit: Lobanov and Zensus (2001). Right: The RadioAstron space VLBI image of the relativistic jet in the blazar 3C 279 reveals parsec-scale helical filaments in the innermost jet region at an angular resolution below 10 microarcseconds, which are undetectable with ground-based arrays alone. The virtual telescope, formed by 23 ground-based antennas including the Effelsberg 100-m telescope and the orbiting RadioAstron spacecraft, spanned a baseline comparable to the Earth-Moon distance (see Fig. 6). Data were correlated at the MPIfR in Bonn. Credit: Fuentes et al. (2023). © NASA/DOE/Fermi LAT Collaboration; VLBA/Jorstad et al.; RadioAstron/Fuentes et al.

RadioAstron enabled a high-resolution survey of compact AGN structures that revealed brightness temperatures far in excess of the Inverse Compton limit across a large sample of sources, providing compelling evidence for extreme Doppler boosting in relativistic jets and demonstrating that sub-10 microarcsecond resolution opens observational parameter space inaccessible to ground-based arrays (e.g., Fuentes et al. 2023; Fig. 7 right). Significant results were also obtained on interstellar masers, pulsar emission structure, and scattering in the interstellar medium. Critically for the black hole imaging goal, however, SMBH observations at the shortest cm wavelengths accessible to RadioAstron were found to be compromised by interstellar scattering and potential self-absorption effects. Interstellar scattering along the line-of-sight to Sgr A* broadens the apparent source at cm wavelengths, smearing out the intrinsic structure that VLBI would otherwise resolve, an effect that decreases steeply with increasing frequency and is greatly reduced at 1.3 mm, where it can be modelled and mitigated (Fish et al. 2014), establishing mm-wave observations at terrestrial baselines as the more tractable path towards imaging SMBH shadows than cm-wave space VLBI.

The achievable resolution of ground-based VLBI is fundamentally limited by baselines of around 10,000 km. Further constraints arise from atmospheric transmission and phase stability: suitable observing conditions at millimetre and sub-millimetre wavelengths are confined to exceptionally dry, high-altitude sites. Few existing radio astronomy facilities are equipped to operate at such sites, and the fraction of time with adequate atmospheric transparency is small. At 1.3 mm, atmospheric phase fluctuations limit the coherence time to only a few seconds, preventing the long integrations needed to accumulate sensitivity on faint sources and making site selection and real-time atmospheric monitoring critical to the success of observations. Nevertheless, at a wavelength of 1.3 mm, ground-based VLBI can achieve an angular resolution of approximately 20 microarcseconds. This is roughly two orders of magnitude finer than that of VLTI/GRAVITY in the infrared regime, and sufficient to resolve the shadows of the two nearest and most massive SMBHs, Sgr A* and M87*.

### Sensitivity and image quality

The sensitivity of mm-VLBI in the 1990s was insufficient for imaging SMBH signatures within feasible observing times, and a coordinated programme of instrumental development was required across multiple parameters. Chief among these was the need for additional collecting area to complement existing facilities such as the IRAM 30-m telescope on Pico Veleta and the Plateau de Bure Interferometer (PdB). The planning of new large mm-wavelength facilities during this period, most notably the Atacama Large Millimeter Array (ALMA) and the Large Millimeter Telescope (LMT) in Mexico, offered a path towards the required sensitivity improvements.

For aperture synthesis arrays such as ALMA or the PdB interferometer (subsequently upgraded and renamed the Northern Extended Millimeter Array, NOEMA), operation in phased-array mode offers two distinct advantages: it maximises the effective collecting area on a given baseline, and it provides a high-sensitivity anchor station for the phase closure technique. The latter allows residual fringe-rates and delays on less sensitive baselines to be derived from detections on sensitive ones, circumventing the need for independent fringe searches on all baselines (e.g., Alef and Porcas 1986). ALMA’s initial configuration did not support phased-array VLBI operations; this capability was subsequently developed and implemented through the ALMA Phasing Project, enabling ALMA’s integration into both GMVA and EHT observing campaigns (Matthews et al. 2018). With 43 of its 54 twelve-metre antennas combined into a single phased element, ALMA provided a collecting area far exceeding that of any other mm-wavelength facility and became the most sensitive station in both the GMVA and EHT arrays, decisively improving detection rates on the weakest baselines.

Beyond optimisation of receivers, telescope surfaces, calibration, and backends, a particularly effective means of increasing array sensitivity for continuum observations is the widening of the recorded bandwidth, a development that demands advances in telescope optics, receivers, and VLBI backends capable of handling data rates of up to 128 Gigabits per second. Recording bandwidths have increased dramatically over the past three decades: from approximately 0.5 GHz in the 1990s to 8 GHz in early EHT campaigns, and most recently to 16 GHz in full polarization.

Sensitivity and baseline coverage are necessary but not sufficient conditions for high-fidelity imaging: the sparse uv-coverage of a heterogeneous array with only a few stations places severe demands on image reconstruction. The EHT images were produced through the independent application of multiple algorithmic approaches, including regularised maximum likelihood methods and variants of the CLEAN algorithm, with consistency across methods serving as a key validation criterion (EHT Collaboration 2019b). Advances in self-calibration, fringe-fitting, and Bayesian imaging techniques have since improved the fidelity and dynamic range of mm-VLBI images (e.g., Kim et al. 2024), enabling the recovery of structural detail inaccessible to earlier methods.

### Organisation and logistics

High-fidelity imaging requires a telescope network capable of simultaneously observing a given source from globally distributed locations, maximising baseline coverage through Earth rotation aperture synthesis. Scheduling such observations imposes stringent constraints: source visibility windows must overlap across sites spanning multiple continents, and seasonal atmospheric statistics at each site must be factored into the observing window selection.

**Fig. 8:**
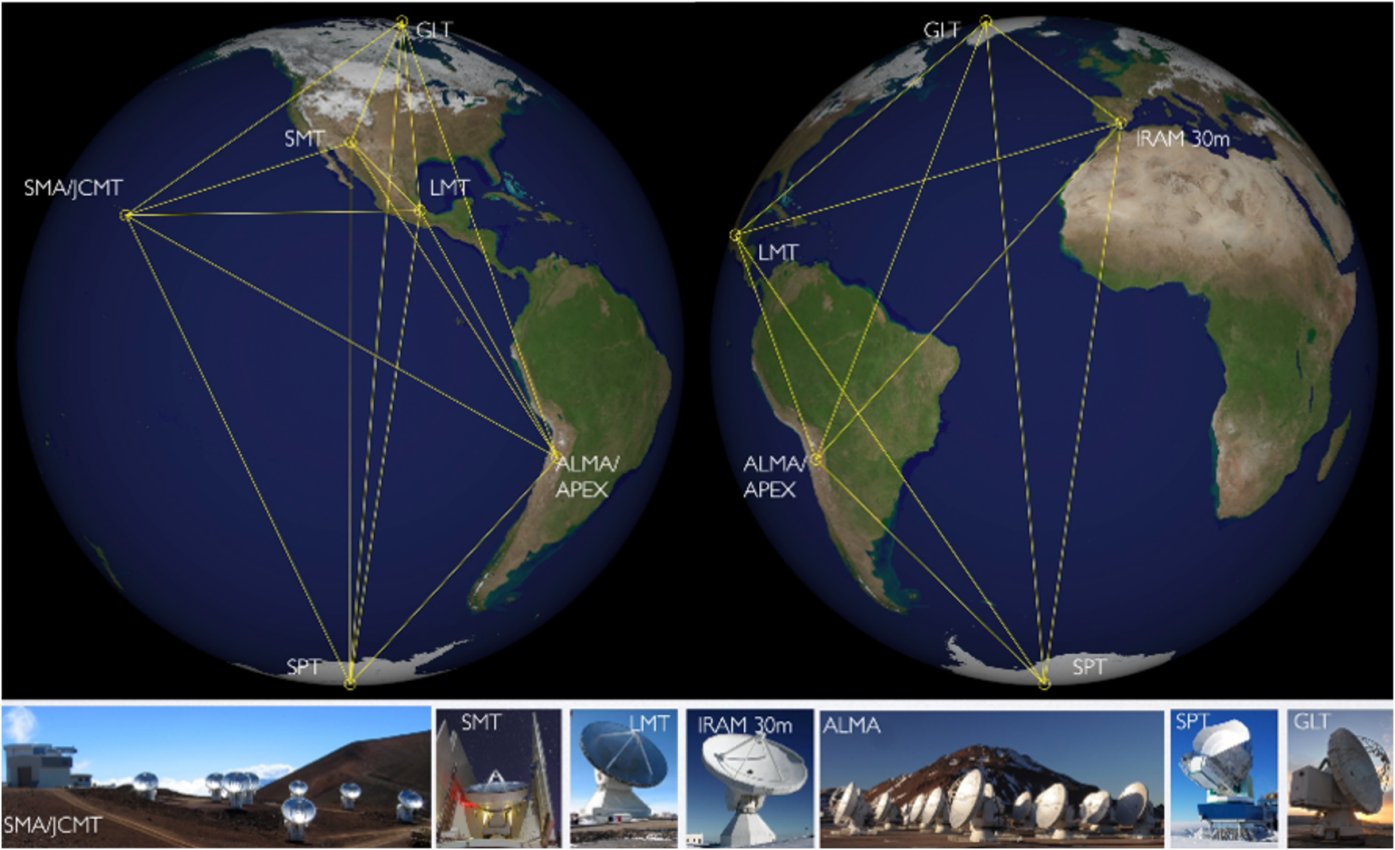
Telescopes participating in the 2018 EHT observing campaign, distributed across the western hemisphere. Earth rotation aperture synthesis provides improved baseline coverage and a cleaner synthesised beam relative to the 2017 array

**Fig. 9:**
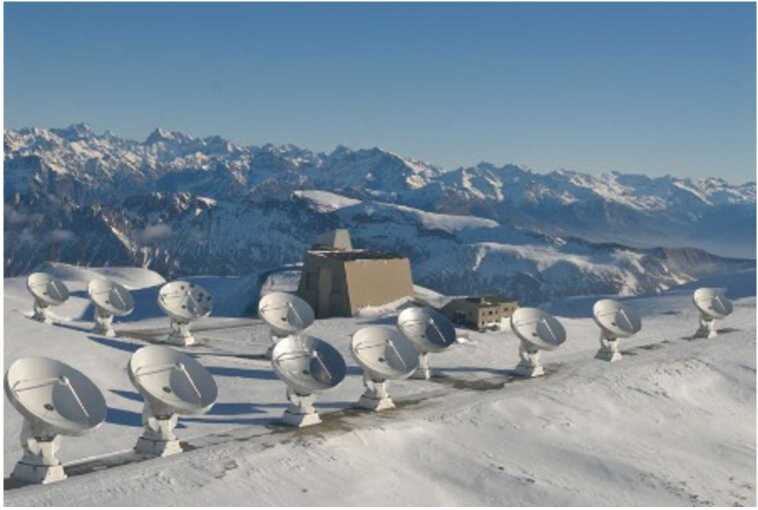
The NOEMA interferometer is one of the major European facilities participating in the EHT programme; together with the IRAM 30-m telescope on Pico Veleta in Spain, the APEX telescope in Chile, and the VLBI correlator in Bonn, it constitutes a major component of the European contribution to the EHT (photo: IRAM)

The 2017 EHT campaign that yielded the first black hole images of M87* and Sgr A* deployed eight facilities: ALMA, the Atacama Pathfinder Experiment (APEX), the James Clerk Maxwell Telescope (JCMT), the Submillimeter Array (SMA), the Submillimeter Telescope (SMT), the Large Millimeter Telescope Alfonso Serrano (LMT), the IRAM 30-m telescope on Pico Veleta, and the South Pole Telescope (SPT) (Fig. 8). Subsequent observations in 2018 extended the array to include the upgraded NOEMA interferometer (Fig. 9), the 12-m telescope on Kitt Peak, and the Greenland Telescope (GLT), with further additions in subsequent campaigns. European facilities played a central role in these observations. The Max Planck Institute for Radio Astronomy contributed through the preparation and operation of APEX at 5,100 m altitude and through VLBI data correlation at the VLBI correlator in Bonn, working in close coordination with colleagues at the IRAM 30-m telescope and with teams at all other participating sites.

Simultaneous favourable atmospheric conditions across all sites cannot be guaranteed, and the success of the 2017 campaign depended critically on an unusually good observing window, a dependence on weather that must be accommodated in the planning of any future campaign. The observations also generated approximately 4,000 Terabytes of data, which required physical shipment by air to the two VLBI correlation centres at Haystack Observatory, USA, and the MPIfR in Bonn, Germany, a logistical necessity given the data volumes involved and the absence of sufficiently fast and reliable network transfer options at the time. Data recorded at the South Pole Telescope presented an additional constraint: since physical access to the site is restricted to the austral summer, transport and correlation of SPT data must await the next access window, typically some six months after the observations.

The EHT array that produced the first direct images of SMBH shadows was not purpose-built but assembled from pre-existing facilities contributed by institutions across multiple countries and funding frameworks. The collaboration ultimately comprised more than 300 researchers spanning observational astronomy, theory, and engineering by the time of the Sgr A* publications. The scale of the undertaking, exceeding the resources of any single nation or continent, necessitated an international institutional framework, a model with substantial precedent in astronomy: most major facilities operate under open-sky policies, and productive international collaborations have been sustained throughout most of the field’s history, including during the Cold War between Western institutions and those in the Soviet Union and China.

Equally critical to the project’s success was the establishment of rigorous governance structures for data reduction and publication. During the imaging phase, independent teams employed different imaging algorithms and were explicitly prevented from sharing intermediate results, ensuring that the published images reflected a convergent outcome independent of any particular analytical approach or preconceived expectation (EHT Collaboration 2019b).

Institutional cooperation within the EHT was formalised through a Memorandum of Understanding establishing the main objectives and conditions for joint decision-making by the EHT Board, which was formed from representatives of the signatory organisations and chaired by the author from the preparatory phase in 2015 to 2023. The Board’s function was to coordinate the priorities of stakeholder organisations and mediate among the interests of the participating partners. Scientific collaboration and operations were directed by Project Director Shepherd Doeleman, who was also a central figure in the North American mm-VLBI effort, supported by a dedicated management team. Scientific input from the membership was channelled through the EHT Science Council, chaired by Heino Falcke. The contributions of the collaboration members, spanning instrumentation, observation, correlation, imaging, and theory, were recognised collectively with the award of the 2020 Breakthrough Prize for Fundamental Physics.

## Future developments

**Fig. 10:**
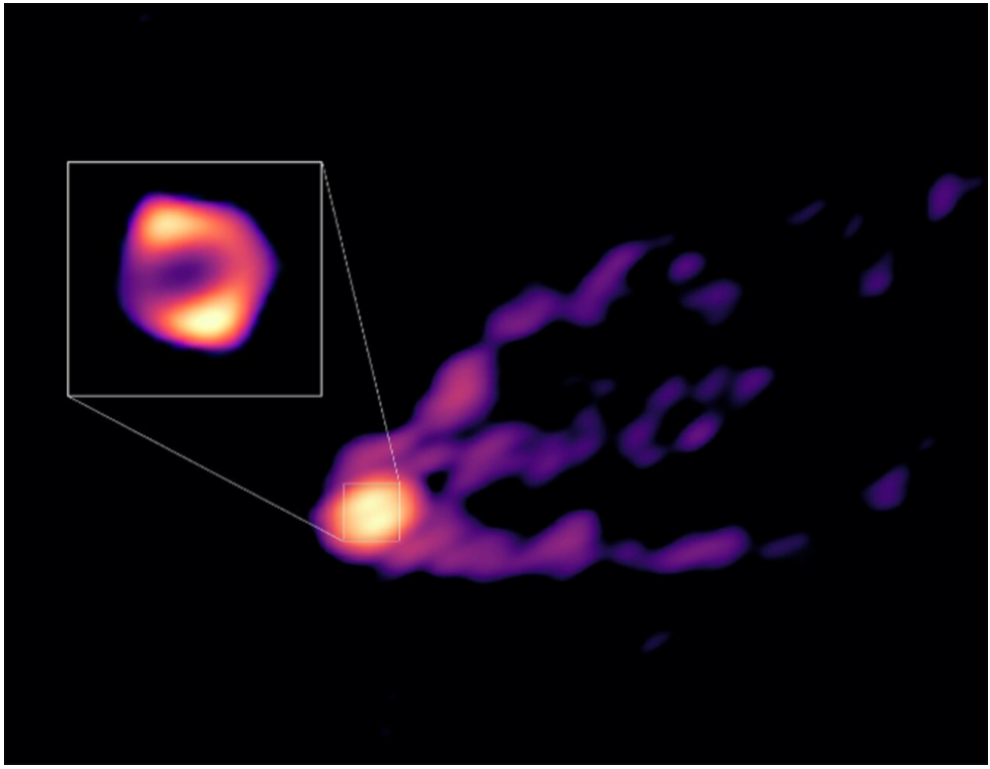
A $\lambda = 3.5\text{ mm}$ GMVA image of M87* obtained in April 2018, showing a ring-like structure approximately 50% larger in diameter than the EHT 1.3 mm image (inset) from April 2017 (Fig. 4). The size difference indicates a substantial contribution from the accretion flow in addition to the gravitationally lensed emission, and the edge-brightened jet is seen to connect directly to the accretion structure (Lu et al. 2023)

The initial EHT publications have generated extensive follow-up activity across the broader research community, with hundreds of independent studies building on the original results to advance understanding of SMBH physics and the properties of the surrounding accretion material and ejected relativistic plasma. Subsequent EHT observations have confirmed the robustness of the original findings. A particularly significant development has been the combination of high-resolution EHT imaging of M87* at 1.3 mm with new 3.5 mm GMVA observations (see Fig. 10), which has enabled systematic study of the structural relationship between the SMBH and the relativistic jet first identified by Heber Curtis over a century ago (Lu et al. 2023; Saurabh et al. 2026).

The role of magnetic fields in jet formation around SMBHs has been investigated through polarimetric observations with both the GMVA and the EHT (EHT Collaboration 2021). Ongoing work pursues this question through polarimetric imaging, the development of new imaging algorithms, and the physical modelling of AGN jets (e.g., Zensus et al. 2024). Coordinated multi-wavelength campaigns, combining mm-VLBI with X-ray, infrared, and gamma-ray observations, are opening a further dimension by linking the radio morphology of the accretion flow and jet to the broadband spectral and variability properties of these systems.

Lower-mass SMBHs, such as Sgr A*, are predicted to exhibit structural variability on timescales shorter than one hour, resulting from the proportionally smaller light-crossing time. This presents a fundamental challenge for static VLBI imaging, which assumes source stationarity over the duration of an observation. The reconstruction of dynamic image sequences, or time-resolved movies of the evolving source structure, requires purpose-developed algorithms capable of handling rapid structural evolution. Such developments would substantially extend both the technical capabilities and the scientific reach of the EHT.

Extending black hole imaging beyond the current two sources requires at least an order-of-magnitude improvement in angular resolution, reaching sub-microarcsecond scales, combined with a corresponding increase in array sensitivity: most candidate SMBHs have flux densities at the milli-Jansky level, several orders of magnitude below the Jansky-level emission of M87* and Sgr A*. Estimates indicate that such a resolution improvement would increase the accessible SMBH population by a comparable factor, extending the observable sample from the local universe to massive systems at cosmological redshifts (Gurvits et al. 2021).

One route towards this goal is a space-borne mm or sub-mm interferometric array, potentially combined with large phased terrestrial facilities such as ALMA and NOEMA to provide additional collecting area and baseline coverage (Gurvits et al. 2022). This approach faces substantial engineering challenges: (1) launch vehicle constraints limit the size of monolithic space antennas, necessitating deployable or phased-array architectures; (2) array element positions must be maintained and monitored to within a fraction of a wavelength at all times; (3) onboard power budgets constrain receiver, backend, and computing performance; and (4) the volume of data to be transmitted to Earth would be considerable. These are formidable but not insurmountable constraints, and the scientific return of high-fidelity images and time-resolved movies of SMBH environments at unprecedented angular resolution would justify the investment.

A nearer-term and cost-effective path towards substantially improved sensitivity is the frequency phase transfer (FPT) technique, which exploits multi-frequency receiver systems operating simultaneously at 86, 230, and 345 GHz to transfer phase solutions from lower to higher frequencies. At mm and sub-mm wavelengths, atmospheric phase noise is the dominant limitation on coherence time and therefore on sensitivity; by deriving phase corrections from the lower-frequency signal, where the atmosphere is more stable, FPT effectively recovers coherence at the higher frequency without requiring independent self-calibration, yielding sensitivity gains potentially approaching an order of magnitude (Dodson et al. 2023). Pioneered by the Korean VLBI Network (Algaba et al. 2015; Dodson et al. 2017; Han et al. 2017), FPT is currently being implemented for the GMVA and is planned for the EHT. Given the cost and extended lead times associated with space missions, FPT offers the prospect of delivering many of the sensitivity benefits anticipated from space VLBI on a timescale considerably shorter than that of a space mission.

The success of the EHT in producing the first images of supermassive black holes rested on three interdependent foundations: (1) access to world-class observational facilities; (2) the application of advanced instrumentation and data analysis techniques, and (3) the effective organisation of a geographically distributed international collaboration. The cooperative culture that characterised the project, spanning observers, instrumentalists, theorists, and engineers across dozens of institutions, was instrumental in translating these foundations into scientific achievement. The images produced by the EHT may in retrospect be seen as a milestone rather than a culmination: the field now confronts technical challenges of a scale comparable to those that defined the early development of mm-VLBI, with the prospect of advancing observational capabilities by orders of magnitude.

## Data Availability

No datasets were generated or analysed during the current study.

## Footnotes

1 The term *shadow* was introduced for the observational context by Falcke et al. (2000) and has been used consistently in EHT publications since. In the earlier theoretical literature, Bardeen (1973) and Luminet (1979) used the term *silhouette* for the same feature. We follow the usage of the EHT Collaboration and adopt *shadow* throughout.

2 The publication of the M87* result in April 2019 was accompanied by a globally coordinated communications campaign; the image received extraordinary international media coverage, appearing prominently across major newspapers, broadcasters, and online publications (Christensen et al. 2019).

3 This paper is based on lectures given at the presentations of the Tycho Brahe Medal of the European Astronomical Society at its meeting in Kraków in 2023, and of the Karl Schwarzschild Medal of the Astronomische Gesellschaft at its meeting in Cologne in 2024.

4 Questions about the accuracy of, and potential biases in, Eddington’s analysis were debated for decades (for an account see Gilmore and Tausch-Pebody 2022) until a re-analysis of the original plates with modern equipment finally confirmed his results (Harvey 1979).

5 Quasars are observed as very compact and bright regions in the centres of extragalactic objects.

6 Luminet’s simulation bears a striking visual resemblance to the rotating black hole *Gargantua*, whose appearance was calculated by Kip Thorne and collaborators (James et al. 2015) for Christopher Nolan’s film *Interstellar* (2014).

7 The resolution in VLBI images is usually given in units of milliarcseconds or microarcseconds. One arcsecond is an angle on the sky of 1/3600 of a degree.

8 Kellermann et al. used the term black hole in their paper referring to Lynden-Bell and the small radio source in the Galactic Centre they had measured, but even that is unlikely to have been the first use of the terms in reference to a compact radio source (K.I. Kellermann, private communication).

9 The shadow of Sgr A* subtends approximately 50 microarcseconds on the sky, comparable to the apparent size of a doughnut on the Moon.
